# Comprehensive Biological Potential, Phytochemical Profiling Using GC-MS and LC-ESI-MS, and In-Silico Assessment of *Strobilanthes glutinosus* Nees: An Important Medicinal Plant

**DOI:** 10.3390/molecules27206885

**Published:** 2022-10-14

**Authors:** Marya Aziz, Saeed Ahmad, Umair Khurshid, Irfan Pervaiz, Arslan Hussain Lodhi, Nasrullah Jan, Sameera Khurshid, Muhammad Adeel Arshad, Mohamed M. Ibrahim, Gaber A. M. Mersal, Fahaad S. Alenazi, Ahmed Awadh Saleh Alamri, Juwairiya Butt, Hammad Saleem, Zeinhom M. El-Bahy

**Affiliations:** 1 Department of Pharmaceutical Chemistry, Faculty of Pharmacy, The Islamia University of Bahawalpur, Bahawalpur 63100, Pakistan; 2 Department of Pharmacy, University of Chenab, Gujrat 50700, Pakistan; 3 Department of Pharmacology, Faculty of Pharmacy, The Islamia University of Bahawalpur, Bahawalpur 63100, Pakistan; 4 Akson College of Pharmacy, Mirpur University of Science and Technology, Mirpur 10250, Pakistan; 5 Department of Pharmaceutics, Faculty of Pharmacy, The Islamia University of Bahawalpur, Bahawalpur 63100, Pakistan; 6 Bahawalpur College of Pharmacy, Bahawalpur Medical and Dental College, Bahawalpur 63100, Pakistan; 7 Institute of Pharmacy, Faculty of Pharmaceutical and Allied Health Sciences, Lahore College for Women University, Lahore 54000, Pakistan; 8 Department of Chemistry, College of Science, Taif University, Taif 21944, Saudi Arabia; 9 Department of Pharmacology, College of Medicine, University of Hail, Hail 55473, Saudi Arabia; 10 Medical Education Unit, College of Medicine, University of Hail, Hail 55473, Saudi Arabia; 11 Medical Services, Ministry of Interior-Security Forces Hospital in Najran, Najran 66256, Saudi Arabia; 12 School of Life Sciences, University of Westminster, 115 New Cavendish Street, London W1W 6UW, UK; 13 Institute of Pharmaceutical Sciences (IPS), University of Veterinary and Animal Sciences (UVAS), Lahore 54000, Pakistan; 14 Department of Chemistry, Faculty of Science, Al-Azhar University, Nasr City 11884, Egypt

**Keywords:** *Strobilanthes glutinosus*, antioxidant, enzyme inhibition, tyrosinase inhibition, GC-MS, LC-ESI-MS, docking

## Abstract

Plants of the genus *Strobilanthes* have notable use in folklore medicines as well as being used for pharmacological purposes. The present work explored the biological predispositions of *Strobilanthes glutinosus* and attempted to accomplish a comprehensive chemical profile through GC-MS of different fractions concerning polarity (chloroform and *n*-butanol) and LC-ESI-MS of methanolic extract by both positive and negative ionization modes. The biological characteristics such as antioxidant potential were assessed by applying six different methods. The potential for clinically relevant enzyme (α-amylase, α-glucosidase, and tyrosinase) inhibition was examined. The DPPH, ABTS, CUPRAC, and FRAP results revealed that the methanol fraction presented efficient results. The phosphomolybdenum assay revealed that the *n*-hexane fraction showed the most efficient results, while maximum metal chelation potential was observed for the chloroform fraction. The GC-MS profiling of *n*-butanol and chloroform fractions revealed the existence of several (110) important compounds presenting different classes (fatty acids, phenols, alkanes, monoterpenes, diterpenes, sesquiterpenoids, and sterols), while LC-ESI-MS tentatively identified the presence of 44 clinically important secondary metabolites. The *n*-hexane fraction exhibited the highest potential against α-amylase (497.98 mm ACAE/g extract) and α-glucosidase (605.85 mm ACAE/g extract). Significant inhibitory activity against tyrosinase enzyme was displayed by fraction. Six of the prevailing compounds from the GC-MS study (lupeol, beta-amyrin, stigmasterol, gamma sitosterol, 9,12-octadecadienoic acid, and *n*-hexadecanoic acid) were modelled against α-glucosidase and α-amylase enzymes along with a comparison of binding affinity to standard acarbose, while three compounds identified through LC-ESI-MS were docked to the mushroom tyrosinase enzyme and presented with significant biding affinities. Thus, it is assumed that *S. glutinosus* demonstrated effective antioxidant and enzyme inhibition prospects with effective bioactive molecules, potentially opening the door to a new application in the field of medicine.

## 1. Introduction

Medicinal plants have shown substantial medicinal and therapeutic benefits, due to which they are becoming important worldwide. Plants have become an object of ample importance in research as well as alternative medicinal therapy [1]. The rapidly increasing population and poverty in the developing world hamper this population from availing of high-priced pharmaceutical products. Medicinal plants are their main source for health care delivery. Around 70–80% of the developing world depends on conventional remedies obtained from medicinal plants [2]. Several novel compounds have been isolated from plants and have demonstrated unique and interesting biological activities [3]. The current research focus is to extract pharmacologically active compounds from natural provenance that can be helpful, particularly in the area of diseases that presently lack an effective medicinal therapy.

There is a major shift of attention from modern medicine to parallel herbal systems, leading to a revival of alternative medicines [4]. According to an estimate, drugs derived from natural sources account for 20–25% of all drugs which are mentioned in the Pharmacopeia. Several medicinal plants are being employed for disease management without any modification [5]. The study of disease progression and induction has shown that oxidative stress is a major causative agent of various diseases. Chronic accumulation of reactive oxygen species causes cellular oxidative stress which ultimately leads to disease progression. Antioxidants of plant origin exhibit great potential; therefore, therapeutic focus has shifted towards the herbal medicine [6]. Phytochemicals possess great antioxidant activity that contributes to the therapeutic efficacy of plants [7].

*Strobilanthes* is a genus belonging to the family Acanthaceae, comprising around 350 species [8]. In this genus, the majority of plants present with anti-inflammatory and wound healing properties. These also show potential antimicrobial, anti-diabetic, and anti-cancer activities. Their extracts have been effective in spider poisoning, influenza epidemic, cerebrospinal meningitis, viral pneumonia, mumps, and acute respiratory syndrome [9]. Even though pharmacological research has described a broad variety of biological activities and chemical properties of the *Strobilanthes* genus (See Supplementary Materials), several species of the genus remain unexplored. *S. glutinosus* is a species that has not been studied scientifically in terms of biological and chemical properties. Only one study regarding the antimicrobial and antioxidant activity of this plant has been conducted in the Department of Botany, Mirpur University of Science and Technology (MUST), Mirpur-10250 (AJK), Pakistan [10].

Therefore, the current work was designed to conduct the chemical analysis to evaluate the bioactive content and GC-MS analysis of different extracts in order to analyze the phytochemical composition. The biological potential was studied by performing antioxidant assays of hydro-methanol extract and *n*-butanol, chloroform, and *n*-hexane fractions of the whole plant of *S. glutinosus*. Additionally, the present work proposed to determine the inhibitory effect of key enzymes (alpha-glucosidase and alpha-amylase) involved in diabetes mellitus along with molecular docking studies to explore any probable interaction between observed secondary metabolites and reported enzyme inhibition results. Molecular docking became an imperative tool for searching for inhibitor interactions at the receptor’s active site. Docking studies, to calculate binding free energy, also reveal the most appropriate confirmation that aids in the development of novel inhibitors against targeted enzymes. In terms of the literature evaluation, this study may be considered the preliminary analysis of the phytochemical composition, antioxidant properties, enzyme inhibition, and molecular docking studies of selected compounds from GC-MS of *S. glutinosus*.

## 2. Results

### 2.1. Phytochemical Composition

**Figure 1:**
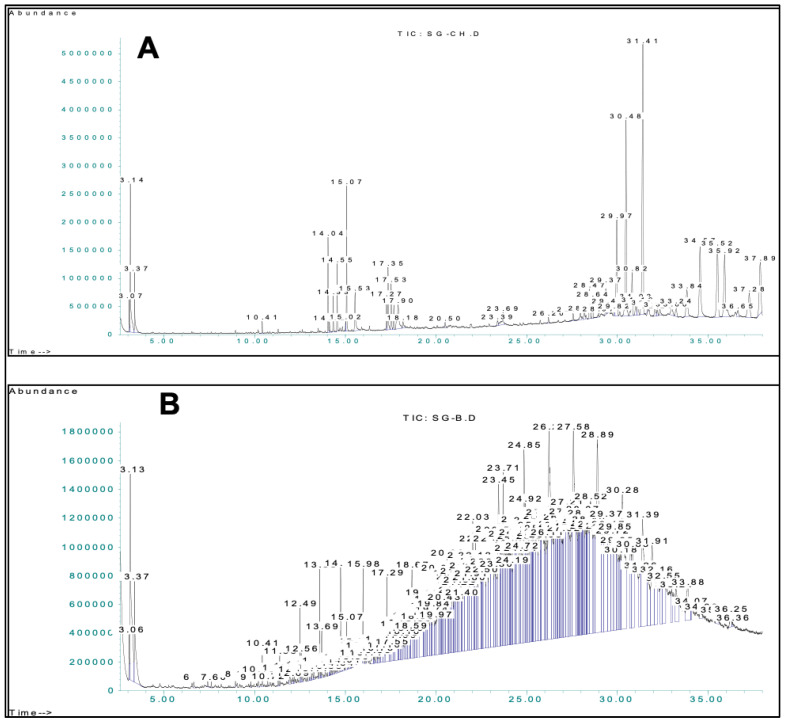
GC-MS chromatograms of chloroform (**A**) and *n*-butanol (**B**) fractions.

In this recent work, two different extracts of *S. glutinosus* were assessed for their bioactive contents via GC-MS, as presented in Table 1 and Table 2 and Figure 1, which enabled the tentative identification of 110 compounds. This GC-MS phytochemical investigation of different extracts of *S. glutinosus* can be considered the first comprehensive study.

To gain a more in-depth insight into the phytochemical composition through the LC-ESI-MS method, we looked into the phytochemicals present in the plant *S. glutinosus* in greater detail. Due to its many benefits, including low solvent consumption, high precision, and accuracy, the hybrid coupled technique is frequently employed for the investigation of phytochemicals derived from plants [11]. 

**Figure 2:**
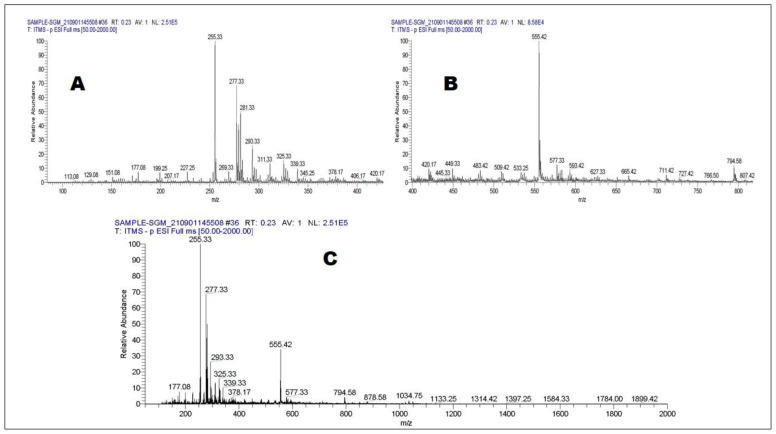
LC-ESI-MS-MS full scan of *Strobilanthes glutinosus* (negative mode) 50–400 (**A**), 50–800 (**B**), and 50–2000 (**C**).

**Figure 3:**
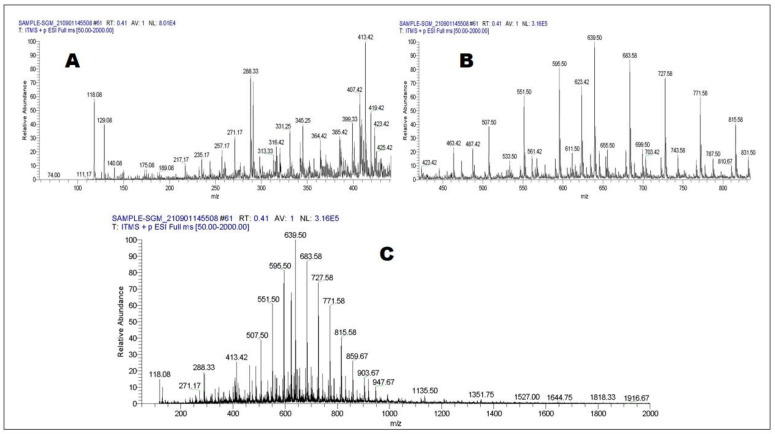
LC-ESI-MS-MS full scan of *Strobilanthes glutinosus* (positive mode) 50–400 (**A**), 50–800 (**B**), and 50–2000 (**C**).

Positive and negative ionizing modes of LC-ESI-MS-MS were used to monitor the profile of secondary metabolites, resulting in the identification of 44 compounds (Table 3 and Table 4). Phenols, phenolic acids, phenolic glycosides, flavonoids, flavonoid glucoside, fatty acids, triterpenoids, lignans, and coumarin are just some of the chemical classes represented by the compounds identified. Figure 2 and Figure 3 represents total ion chromatograms of both negative and positive ionization modes.

### 2.2. Antioxidant Assays

In the present study, six different methods (DPPH, ABTS, FRAP, CUPRAC, phosphomolybdenum, and metal chelating assays) were used to determine the antioxidant potential of *S. glutinosus*, and Table 5 furnishes the results of the study. 

### 2.3. In Vitro Enzyme Inhibition Activity

The studied plant extracts were tested against different enzymes, including α-amylase, α-glucosidase, and tyrosinase. The standard used for α-amylase and α-glucosidase was acarbose, and the results were presented in mmol ACAE/g extract. Kojic acid was used as the standard for tyrosinase enzyme, with results being presented in mg KAE/g extract. The inhibitory potential of plant extract/fractions against all three enzymes is displayed in Table 6. Maximum percentage inhibition against α-amylase and α-glucosidase was displayed by chloroform fraction (501.407 ± 2.982 and 605.854 ± 6.252 mmol ACAE/g extract), respectively. While methanolic extract exhibited the highest potential against tyrosinase enzyme (9.86 ± 1.41 mg KAE/g extract).

### 2.4. In Silico Analysis

**Figure 4:**
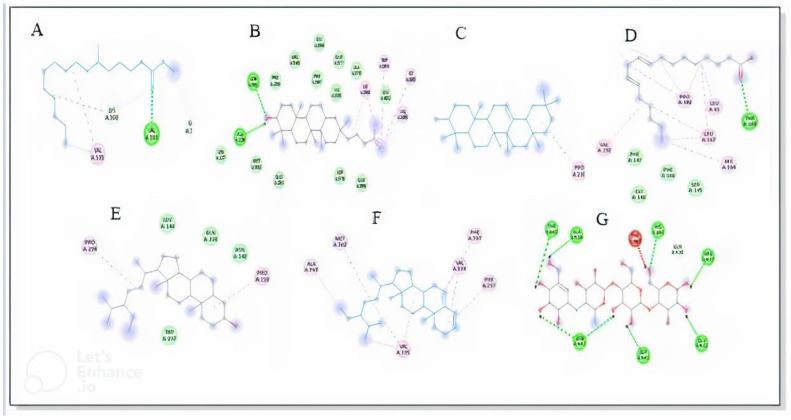
Enzyme α-glucosidase and ligands interaction. Hexadecanoic acid (**A**), Lupeol (**B**), β-Amyrin (**C**), Octadecadienoic acid (**D**), Sitosterol (**E**), Stigmasterol (**F**), Acarbose (standard) (**G**).

**Figure 5:**
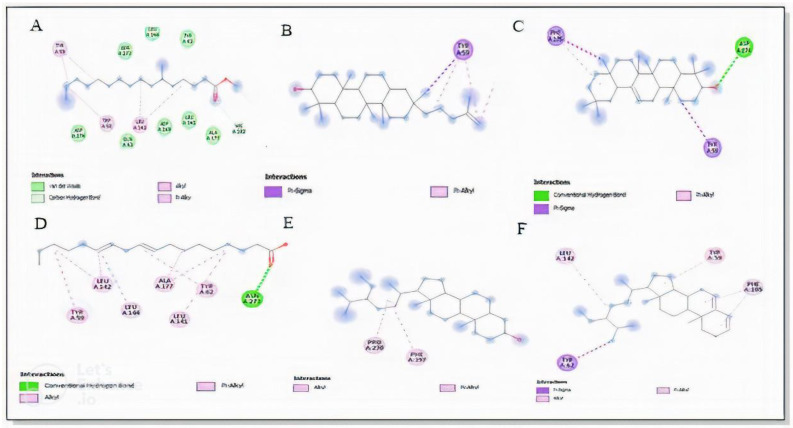
Enzyme α-amylase and ligands interaction. Hexadecanoic acid (**A**), Lupeol (**B**), β-Amyrin (**C**), Octadecadienoic acid (**D**), Sitosterol (**E**), Stigmasterol (**F**).

A total of 29 compounds identified in GC-MS analysis of the chloroform fraction were docked, and six of them were selected based on their binding affinities along with the standard compound acarbose against the receptor α-glucosidase and α-amylase enzymes. PubChem, the drug database, was used for downloading the 3D structure of ligand molecules. Beta-amyrin, sitosterol, stigmasterol, and lupeol were identified to be the most suitable ligands, with significant binding affinities. Our results indicated that beta amyrin had the highest binding affinity with the α glucosidase enzyme, with a docking score of –8.4 kcal/mol, followed by stigmasterol (−7.5 kcal/mol), sitosterol (−7.5 kcal/mol), lupeol (−6.9 kcal/mol), 9,12-octadecadienoic acid (−4.1 kcal/mol), and *n*-hexadecanoic acid (−3.5 kcal/mol), presented in Table 7 and Figure 4 and Figure 5. Beta amyrin, sitosterol, and stigmasterol presented the highest binding affinity versus α glucosidase enzyme, with a binding energy of −8.4 and −7.5 kcal/mol, respectively, ranking higher in comparison to standard drug acarbose (−6.6 kcal/mol).

**Figure 6:**
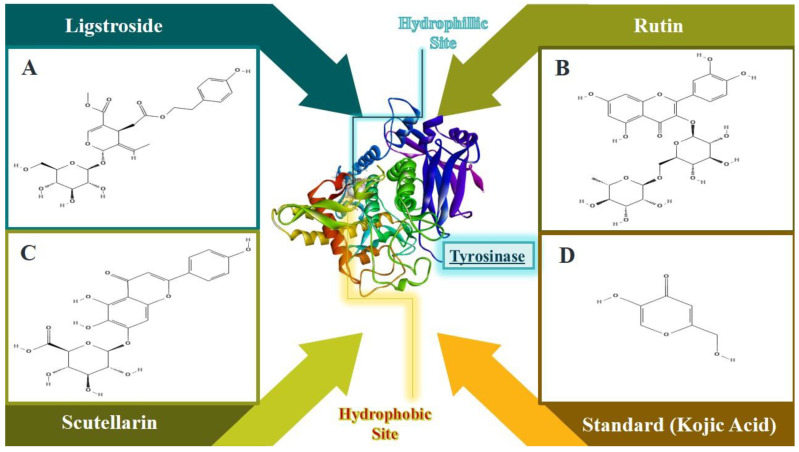
Molecular docking of selected ligands with tyrosinase enzyme. (**A**) Ligstroside, (**B**) Rutin, (**C**) Scutellarin, and (**D**) Kojic acid.

**Figure 7:**
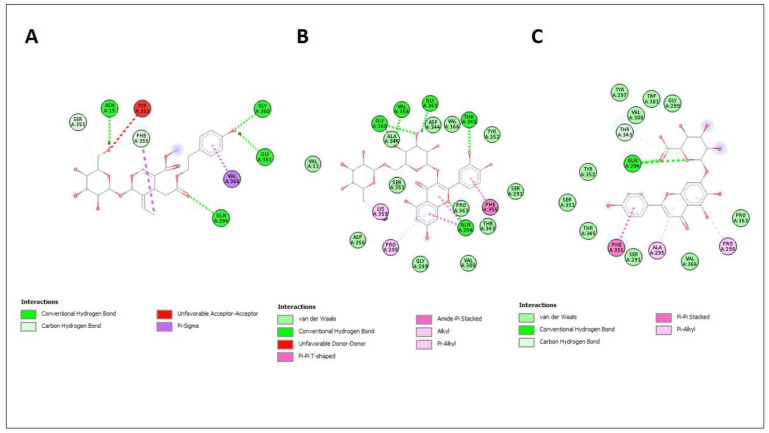
Enzyme tyrosinase and ligands interaction. Lingstroside (**A**), Rutin (**B**), Scutellarin (**C**).

Molecular docking on three key compounds (Lingstroside, Rutin, and Scutellarin) identified from the methanolic extract using LC-ESI-MS (Figure 6) was performed against mushroom tyrosinase enzyme. As a reference drug for such conditions, kojic acid was also included in the assay. The binding affinities and amino acid interactions are presented in Table 8 and Figure 7.

## 3. Discussion

Bioactive chemicals such as those found in plants are crucial to human health because they stimulate cell division and repair, two processes essential to being healthy as a whole [12]. There is evidence in the literature that indicates *S. glutinosus* methanol extract has significant flavonoid and phenolic levels [10]. Overall, 29 and 81 major compounds were identified from chloroform and *n*-butanol fractions, respectively. From 110 compounds, *n*-hexadecanoic acid, 9,12-Octadecadienoic acid (2,2)-, methyl ester, linoelaidic acid, 11,13-dimethy1-12-tetradecane-1-olacetate, heptadecanal, alpha-tocospiro A, alpha-tocospiro B, dl- stigmasterol, gamma–sitosterol, lup-20(29)-en-3-one, lupeol, and linoleic acid were identified as major bioactive compounds from the whole-plant extract. Lupeol (sterol) is one of the major compounds detected in high concentrations in the GC-MS study. Lupeol is found naturally in edible fruits and vegetables and is reported to have antioxidant, anti-diabetic, hepatoprotective, anti-inflammatory, anti-protozoal, anti-microbial, anti-proliferative, and cholesterol-lowering effects [13]. Another compound having a reported anticancer activity and antioxidant effect is 9, 12-Octadecenoic acid methyl ester (Z, Z), fatty acid methyl ester [14].

The pharmacological benefits of flavonoids and phenols are well-documented. Many studies have shown that phenols and flavonoids are effective antioxidants, anti-inflammatory agents, and enzyme inhibitors, with significant clinical uses [15,16,17,18]. In the present study (LC-ESI-MS), a total of 14 flavonoids were identified, 6 in negative mode and 8 in positive mode of ionization. Deprotonated molecules [M-H]^−^ were observed in Daidzein (Rt = 12.25 min), Hispidulin (Rt = 12.46), 8-Prenylnaringenin (Rt = 2.63), (−), Epicatechin 3-O-gallate (Rt = 15.01), Myricetin 3-O-arabinoside (Rt = 15.99), and Luteolin 7- rutinoside (Rt = 17.05), while Apigenin (Rt = 0.57 min), Eriodictyol (Rt = 3.43), 5-OH liquiritin (Rt = 6.21), scutellarin (Rt = 6.42), and Rutin (Rt = 7.87) displayed ions’ positive mode of ionisation. Similarly, 10 phenols and their derivatives were tentatively identified in both the negative and positive mode of ionisation analysis. Syringic acid (Rt = 11.91), p-coumaryl malic acid (Rt = 2.21), catechin (Rt = 2.37), and gingerol (Rt = 13.32) are the phenols found, and all of them showed deprotonated molecules [M-H]^−^. In the present chromatographic analysis of the *S. glutinosus* methanol fraction, three phenolic acids, caffeoyl tartaric acid (Rt = 13.40), *p*-coumaric acid hexoside (Rt = 13.61), and gentesic acid (Rt = 2.28), were tentatively identified. Additionally, catechin was detected in the positive ionisation mode, where it showed a peak at *m*/*z* 291.00. A single phenolic glycoside, gallic acid hexoside (Rt = 3.98), was tentatively identified in the positive mode of ionization by displaying the protonated molecule [M+H]^+^ at 331.50. In the process, two lignans, sesamolinol (Rt = 14.09) and lariciresinol-sesquilignan (Rt = 16.34), were isolated in the negative mode of the analysis. Emmotin A, a terpenoid (Rt = 12.70), was detected in the negative mode, as were campesterol (Rt = 14.49) and beta amyrin (Rt = 14.71), while tocopherol (Rt = 5.90) was detected in the positive mode.

The oxidative stress caused by reactive oxygen species has been linked to the pathogenesis of a wide variety of degenerative illnesses [19]. Endogenous antioxidants with exogenous antioxidants mostly derived from plants prevent the oxidative stress [20] Plants are the main source of antioxidant compounds. Consequently, for oxidant-induced diseases, research has been focused on plants [19]. Antioxidant effects cannot be established by using a single method since plant extracts contain a large number of chemicals that comprise antioxidant activity with a varied mechanism of action [21]. For these reasons, different methods were applied to analyze the antioxidant activity of plant extracts. 

The hydro-methanol extract of *S. glutinosus* expressed the highest value in the DPPH assay (56.21 mg TE/g extract) and ABTS assay (63.46 mg TE/g extract) TE/g extract. The *n*-butanol and chloroform fractions shared almost similar results in both assays. whereas the lowest effect was measured towards the *n*-hexane fraction (DPPH: 9.80 mg TE/g extract) and (ABTS: 12.76 mg TE/g extract).

The extract’s antioxidant activity is also significant because of its reducing power. By reducing the ferric tripridyltriazine complex to the ferrous complex at low pH, the FRAP assay measures the antioxidant’s capacity to contribute electrons to reduce ferric ions. The hydro-methanol extract presented higher reducing power (87.12 mg TE/g extract), while the *n*-hexane fraction exhibited the lowest reducing potential (34.97 mg TE/g extract). The *n*-butanol and chloroform fractions shared almost similar results, i.e., 75.09 and 65.00 mg TE/g extract, respectively. In the results for the CUPRAC assay, the hydro-methanol extract exhibited higher values (245.11 mg TE/g extract) following the order from hydro-methanol > *n*-butanol > chloroform > *n*-hexane. There was a link between free radical scavenging assays and reducing power assays, suggesting that the bioactive content results verified the increased amount of phenolic and flavonoid components in methanol and butanol extracts [22].

Furthermore, a phosphomolybdenum assay was used to evaluate the total antioxidant capacity, and the findings are furnished in Table 2. The *n*-hexane fraction of *S. glutinosus* exhibited the highest total antioxidant capacity (119.58 mg TE/g extract), while hydro-methanol extract and chloroform fraction exhibited considerable total antioxidant capacity potential, with values of 96.01 and 60.69 mg TE/g extract, respectively. The *n*-butanol fraction was the least active fraction for this assay (6.54 mg Trolox equivalent/g). The presence of non-phenolic compounds with chelating properties among phytoconstituents is consistent with other findings reported by [23]. The GC-MS study of *S. glutinosus* presented the compounds thymol, lupeol, alpha-tocopherol, and squalene, having antioxidant activity reported by [24,25,26], which justifies the results.

In developing nations, where Type 2 diabetes mellitus accounts for 90% of all cases, the prevalence of diabetes mellitus is anticipated to more than double, from 171 million in 2000 to 300 million by 2025 [27]. Popular antidiabetic drugs such as acarbose, voglibose, and miglitol all work by inhibiting alpha-amylase and alpha-glucosidase enzymes, resulting in lower blood glucose levels. However, these drugs have undesirable side effects, including toxicity to the liver and gastrointestinal issues, when used long-term [28]. Consequently, there is a demand for novel alpha-glucosidase and alpha-amylase inhibitors derived from natural origins, particularly from herbs and plants that produce no unpleasant or undesirable side effects in diabetic patients. 

The results of alpha-amylase, alpha-glucosidase, and tyrosinase inhibition assays of different fractions of *S. glutinosus* are presented in Table 6. Among the tested extracts, chloroform fraction was most efficient against α-amylase (501.407 ± 2.98 mmol ACAE/g extract) and α-glucosidase enzyme (605.854 ± 6.252 mmol ACAE/g extract). Likewise, the hydro-methanol extract was also noticeably active against both (amylase and glucosidase) enzymes, with the values of 166.758 ± 1.721 and 294.195 ± 3.036 mmol ACAE/g extract, respectively. The evaluated anti-diabetic potential of *S. glutinosus* complies with the earlier reports as described in [29], an in vivo assay of *S. cuspidata* to evaluate the anti-diabetic potential and the isolated compounds. Lupein, (3-Hydroxy-4-methoxy phenyl) cinnamic acid and stigmasterol exhibited confirmed antidiabetic potential by inhibiting α amylase enzyme [29]. 

Melanin biosynthesis, also called melanogenesis, is a physiological process that is catalysed in humans by the enzyme tyrosinase [30]. Tyrosinase inhibitors may be useful for treating dermatological disorders associated with melanin hyperpigmentation [31], as they work by reducing the activity of tyrosinase, an enzyme that is responsible for the production of melanin. Tyrosinase inhibition can also be useful for the food industry. However, preventing tyrosinase activity is ideal for preserving the freshness of fruits and vegetables for a longer period of time. The methanolic extract showed prominent activity against the tyrosinase enzyme with a value of 9.86 ± 1.41 mg KAE/g extract. The tyrosinase inhibition results of *S. glutinosus* extracts were ordered as follows: methanol > *n*-butanol > chloroform > *n*-hexane. Studies have shown that various phenolics and flavonoids (as revealed via LC-ESI-MS of *S. glutinosus* methanol extract) have anti-tyrosinase potenial.

To accurately anticipate the ligand–target binding energy and to offer an understanding of the molecular-based mechanism of biological processes that ligands produced, computational techniques have been successfully employed in the pharmaceutical and nutraceutical industries. More information on how physiologically active chemicals can bind to certain enzymes can be gleaned through molecular docking studies [32]. The ligand molecules were lupeol, beta amyrin, stigmasterol, gamma sitosterol, 9,12-octadecadienoic acid, and *n*-hexadecanoic acid. The energy and stability of the conformer were then minimized before docking to obtain the lowest energy and a more stable conformer. The binding of certain proteins with ligands promotes the efficiency of biological activity. The analysis of the protein interaction with the ligand is an important element for drug delivery and molecular pathways information. The docking outcome within each ligand to the receptor was evaluated using the docking energy (Kcal/mol) as well as the binding of every ligand with active domains of α-glucosidase and α-amylase. 

For the mushroom tyrosinase enzyme, rutin had the highest binding affinity (−8.9 kJ/mol), followed by scutellarin (−8.6 kJ/mol) and Lingstroside (−8.0 kJ/mol). The reference material (kojic acid) had a binding affinity of −5.3 kilojoules per mole. In contrast to ligands binding via traditional hydrogen bonding, those engaging via van der Waals forces and other weak intermolecular forces were discovered to have higher binding affinities. In the 2D docking data, van der Waals force interactions are substantially more prevalent than conventional hydrogen bonds between amino acids. This proved that our ligands have greater enzyme binding affinities than those previously reported.

## 4. Materials and Methods

### 4.1. Plant Collection and Extraction

*S. glutinosus* (whole plant) were collected from Abbottabad when plants were fully grown. *S. glutinosus* plant was identified by Dr. Sarwer from Islamia University, Bahawalpur, Pakistan and the specimen was placed in the Department of Botany’s Herbarium. The collected whole plant (08 kg) was extracted with 80% hydro alcoholic solvent (methanol and water with (80:20)) for 7 days with occasional shaking. The filtration was performed with filter paper and the solvent evaporation was conducted under vacuum through a rotary evaporator. The extract was fractionated with different solvents from low polarity to high polarity (*n*-hexane, chloroform, and *n*-butanol). The extracts are stored at the appropriate temperature (until required for further use).

### 4.2. Phytochemical Analysis

#### 4.2.1. GC-MS Analysis

The equipment for GC-MS was Agilent, series 6890 and the detector was Hewlett Packard, 5973. Separations were attained by a coloumnHP-5MS column (length30 m × diameter 250 μm × thickness of film 0.25 μm). An electron ionization system with high energy electrons (70 eV) was utilized for spectroscopic detection by GC-MS. The temperature of the injector was 220 ± 0.2 °C and the transfer line 240 °C. The temperature of the oven was programmed from 60 °C to 246 °C at 3 °C/min. Pure helium gas was passed as a carrier at 1.02 mL/min at 210 °C. Prepared extracts, 1.0 µL diluted with methanol as a solvent, were injected at 250 °C in a split less method. The early temperature was positioned at 50–150 °C with a rising rate of 3 °C/min and held for 10 min. Finally, the temperature was amplified to 300 °C at a rate of 10 °C/min [33]. Detection was completed using a full scan mode between 35 to 600 *m*/*z* and with a gain factor of 5. The NIST 2011, Library was used for bioactive compounds identification.

#### 4.2.2. LC-ESI-MS Analysis

Crude methanol extracts showing significantly increased antioxidant activities were also investigated using LC-ESI-MS-MS of model LTQ XL (Thermo Scientific, Waltham, MA, USA). The electron spray ionisation direct inject method was used for both negative and positive mode identification. The capillary temperature was kept constant at 290 °C. The voltage applied to the capillary was 4.7 kilovolts. We kept the sample flow rate constant at 7.8 μL/min. The 50–2000 *m*/*z* mass range was successfully controlled. The nature of the parent molecular ion dictated the energy range (5–30) over which collisions were induced for fragmentation during MS/MS. All of the samples came from the same place and under the same conditions. The ESI-MS/MS data were analysed by hand using specialised software (Xcalibur 2.0.7). The structure was elucidated using ChemDraw Ultra 12.0, and the results were correlated with previously published studies [34].

### 4.3. Antioxidant Activity

#### 4.3.1. Radical Scavenging Activity

The DPPH and ABTS tests were used to assess the radical scavenging ability for extracts in accordance with the previously described procedure [35]. For the DPPH assay, whole-plant extract of 4 fractions each of 0.5 mL was added to 4 mL of DPPH (0.267 mM). The absorbance of different extracts was calculated at mg 517 nm. Trolox equivalents per gram of dry extract (mg TE/g extract) were used to calculate the findings. The ABTS assay was carried out by incubating the 2 mL of ABTS solution (2.5 mM), 0.5 mL of each extract solution, and 2.45 mM potassium persulfate (equal volume) for 30 min in the dark and at 734 nm absorbance was measured. Trolox equivalents per gram of dry extract (mg TE/g extract) were used to calculate the findings.

#### 4.3.2. Reducing Power Assays

CUPRAC and FRAP assays have been used to determine the reductive potential of *S. glutinosis* whole-plant extracts, in accordance with previously described procedures [36]. For cupric ion reducing activity (CUPRAC assay), each extract (0.5 mL) was mixed with 10 mM CuCl_2_ (1 mL), 7.5 (mM) neocuproine (1 mL), and 1M NH_4_Ac buffer at pH 7.0 (1 mL). The absorbance was estimated at 450 nm after 30 min of incubation at room temperature. In the same manner, the blank sample was also prepared, other than the extract. The measurement unit was milligrams of Trolox equivalents per gram of dry extract (mg TE/g extract). For ferric-reducing antioxidant power (FRAP), 0.5 mL of extract solution in methanol (10 mg/10 mL) was vortexed with 2 mL of a FRAP reagent, and 225 µL of water was added and warmed at 37 °C. Then, the absorbance was read at 593 nm. The FRAP values were measured as milligrams of Trolox equivalents per gram of dry extract (mg TE/g extract).

#### 4.3.3. Total Antioxidant Activity

The total antioxidant capacity of the extracts obtained from *S. glutinosus* was evaluated by using the phosphomolybdenum method in agreement with the previously described procedure [37], with few modifications. First, 0.5 mL of extract solutions with methanol (1 mg/1 mL) was added to reagent mixture consisting 0.6 M sulfuric acid (0.6 M), sodium phosphate (28 mM), and ammonium molybdate (4 mM). The mixture was incubated for 90 min at 95 °C and absorbances were recorded at 695 nm beside a blank sample having 0.5 mL methanol with a 3 mL reagent mixture. The measurement unit was milligrams of Trolox per gram of dry extract.

#### 4.3.4. Metal Chelating Activity

The metal chelating assay was performed in accordance with the previously described procedure [17], with some modifications. The fraction solution with methanol (0.5 mL) was added to 0.05 mL FeCl_2_ (2 mM). The reaction was in progress, using 0.2 mL ferrozine (5 mM). Likewise, a blank sample was prepared without ferrozine. The absorbances of all fractions were recorded after incubation at room temperature for 10 min at 562 nm. The milligrams of EDTA equivalents per gram of dry extract (mg EDTAE/g extract) were used for measurement.

### 4.4. Enzyme Inhibitory Activities

The capacity of the different extract/fractions obtained from *S. glutinosus* to inhibit the α-amylase and α-glucosidase was described by the previously outlined procedure [17]. For α-amylase inhibition assay, the reaction mixture containing the different fractions of extract solution (0.5 mL) and alpha-amylase solution (10 μ/mL, 1 mL) with phosphate buffer (6 mM sodium chloride (pH 6.9) was put into the starch solution (0.05%, 0.5 mL). HCl (0.5 mL, 1 M) and 1 mL of iodine-potassium iodide solution were added to stop the reaction. The reaction mixtures were incubated for 10 min at 37 °C. The blank was prepared with the same procedure without the extract. The absorbance readings were noted at 630 nm. Milligrams of acarbose equivalents per gram of dry extract were the measuring unit.

The α-glucosidase inhibition assay was followed with the addition of 0.5 mL of different fraction solutions in equivalent concentrations of 0.5 mL glutathione (0.5 mg/mL) with α-glucosidase solution (0.2 u/mL) in phosphate buffer (pH 6.8) and PNPG (10 mM). After 15 min, the reaction was stopped with the addition of 0.5 mL of sodium carbonate solution (0.2 M). The absorbance readings were noted at 400 nm. The results were expressed in milligrams of acarbose equivalents per gram of dry extract (ACAEs/g extract). For tyrosinase inhibition, plant extracts were tested for their ability to inhibit the enzyme by the standard method already reported [38]. Kojic acid equivalents (KAE/g) were used to quantify the inhibitory effects on tyrosinase enzyme.

### 4.5. In Silico Analysis

Computer-aided molecular modeling was used to examine the conformational relationship between the compound and enzyme. The drug database was used for downloading the 3D structure of ligand molecules. Crystal structures of α-glucosidase (3F5L), α- amylase (3BC9), and tyrosinase (5M6B) were downloaded from the RCSB PDB protein data bank http://www.rcsb.org/pdb (accessed on 5 January 2022). The structure file (XML and PDB format) was converted to PDBQT format using Open Babel 2.4.1. AutoDock Vina, which was offered by the server, was used to determine the required hydrogen atoms. Auto grid software with connected grid data was employed for blind docking. The initial position, orientation, and torsions were all randomized (Santos et al., 2016). The energy and stability of the conformer were then minimized before docking to obtain the lowest energy and a more stable conformer. The extracted data were compared and validated with the experimental data for α-glucosidase and α-amylase complexed with acarbose ligand [39]. According to a recent study, most of the protein–ligand bindings are based on hydrogen bonds, ionic interactions, and van der Waals interactions. As a result, they were specifically targeted in this work by using Biovia/Discovery Studio 2021.

### 4.6. Statistical Analysis

The average of three similar experiments was used to calculate the effects, which were represented through the average ± SD of value. The results were analyzed using one-way ANOVA from SPSS v. 17.0. Statical significance was considered as the value of *p* < 0.05.

## 5. Conclusions

The present research has compared the biological properties and chemical characterization of different polarity solvent extract/fractions of *S. glutinosus*. The GC-MS analysis of chloroform and *n*-butanol fractions was performed and compared to provide more detail about the chemical profile. Fatty acids, phenols, monoterpenes, diterpenes, and sesquiterpenoids were identified as the key classes. In terms of inhibitory effects and in silico studies towards tyrosinase, α-amylase, and α-glucosidase, all extracts demonstrated different capabilities against these enzymes, and in silico studies of six selected compounds from GC-MS also provide the basis for ant-diabetic potential. Furthermore, molecular modelling of three flavonoids identified through LC-ESI-MS were docked to the tyrosinase enzyme to validate the plant’s tyrosinase inhibition potential. Based on our observation, *S. glutinosus* could be recognized as a promising potent biological agent possessing antioxidant, anti-diabetic, and anti-melanogenic properties. The high number of flavonoids and phenols identified in LC-ESI-MS analysis of the plant *S. glutinosus* in the present study may account for its powerful antioxidant and enzyme inhibition potential. However, further research concerning isolation, identification, and description of its bioactive compounds is essential to discover its potential applications in the field of medicine.

## Figures and Tables

**Table 1:** GC-MS analysis of chloroform fraction of *S. glutinosus*.

Sr.	RT	% Area	Name of Compound	Mol. Weight	Mol. Formula	Chem. Class
1	3.07	0.69	Ethylbenzene	106	C_8_H_10_	Alkylbenzene
2	3.14	4.14	Benzene, 1,3-dimethyl-	160	C_8_H_4_C_l6_	Alkylbenzene
3	3.37	1.75	p-Xylene	106.16	C_8_H_10_	Hydrocarbon
4	10.41	0.27	Phenol, 2,5-bis(1,1-dimethylether	206.32	C_14_H_22_O	Phenol
5	14.12	0.29	2-Pentadecanone, 6,10,14-trimet, …	268.5	C_18_H_36_O	Sesquiterpenoids
6	14.33	0.90	9-Octadecene	252.5	C_18_H_36_	Hydrocarbon
7	15.02	0.27	2-Cyclopenten-1-one, 2-pentyl-	152.3	C_10_H_16_O	Cyclic ketones
8	15.07	3.57	Hexadecanoic acid, methyl ester	270.5	C_17_H_34_O_2_	Fatty acid
9	17.27	0.94	9,12-Octadecadienoic acid, meth, …	294.5	C_19_H_34_O_2_	Fatty acid
10	17.90	2.40	(R)-(-)-14-Methyl-8-hexadecyn-1-ol	252.4	C_17_H_32_O	Hydrocarbon
11	18.18	0.41	2-Piperidinone, N-[4-bromo-n-bu, …	234.1	C_9_H_16_BrNO	Delta-lactams
12	27.56	0.34	Pyridine-3-carboxamide, oxime, …	137.4	C_6_H_7_ N_3_O	Oxime
13	27.97	0.44	2-Ethylacridine	207.2	C_15_H_13_N	Acridine
14	28.18	0.47	Cyclotrisiloxane, hexamethyl-	222.4	C_6_H_18_O_3_Si_3_	Organosilicon
15	28.47	1.33	Eicosane	282.5	C_20_H_42_	Alkane
16	28.64	0.74	Cholesta-6,22,24-triene, 4,4-di, …	394.7	C_29_H_46_	Sterol
17	29.37	1.18	1,3,5-Trisilacyclohexane, 1,1-d, …	339.0	C_3_H_6_Cl_6_Si_3_	Hetrocyclic
18	29.97	5.53	Cholest-5-en-3-ol (3.beta.)-, c, …	386.7	C_27_H_46_O	Cholesterol
19	30.82	2.42	Ergosta-4,6,22-trien-3.beta.-ol	396.6	C_28_H_44_O	Sterol
20	31.02	0.85	Phenylacetic acid, 2-(1-adamant, …	298.4	C_2o_H_26_O_2_	Ethyl ester
21	31.12	0.74	Benz[b]-1,4-oxazepine-4(5H)-thi, …	207.2	C_11_H_13_NOS	Alkyl benzene
22	32.35	0.43	2,4-Cyclohexadien-1-one, 3,5-bi, …	184.1	C_12_H_8_O_2_	Cyclohexadien
23	33.00	0.77	1H-Indole, 1-methyl-2-phenyl-	207.2	C_15_H_13_N	Phenyl indole
24	33.24	0.76	1-Bromoeicosane	361.4	C_20_H_41_Br	Alkane
25	33.84	2.29	Campesterol	400.7	C_28_H_48_O	Sterol
26	35.92	5.74	Stigmasterol, 22,23-dihydro-	412.7	C_29_H_48_O	Sterol
27	36.65	0.46	beta.-Amyrin	426.7	C_30_H_50_O	Triterpenoid
28	37.28	2.52	Lup-20(29)-en-3-one	424.7	C_30_H_48_O	Triterpenoid
29	37.89	4.58	Lupeol	426.7	C_30_H_50_O	Triterpenoid

**Table 2:** GC-MS analysis of *n*-butanol fraction of *S. glutinosus*.

Sr.	RT	% Area	Name of Compound	Mol. Weight	Mol. Formula	Chem. Class
1	3.06	0.13	Ethylbenzene	106.1	C_8_H_10_	Ar. hydrocarbon
2	3.13	1.31	*p*-Xylene	106.1	C_8_H_10_	Ar. hydrocarbon
3	3.37	0.64	*o*-Xylene	106.1	C_8_H_10_	Ar. hydrocarbon
4	6.63	0.03	*m*-Mentha-4,8-diene, (1S,3S)-(+)-	136.2	C_10_H_16_	Ar. hydrocarbon
5	7.40	0.01	1H-Inden-1-one, 2,3-dihydro-3,4, …	174.2	C_12_H_14_O	Indanones
6	7.60	0.01	Decane, 3,8-dimethyl-	170.3	C_12_H_26_	Ali. hydrocarbon
7	8.94	0.02	1-Tetradecene	196.3	C_14_H_28_	Ali. hydrocarbon
8	9.77	0.02	Nonadecane	268.5	C_19_H_40_	Ali. hydrocarbon
9	10.19	0.05	Pentacosane	352.7	C_20_H_52_	Ali. hydrocarbon
10	10.42	0.09	Phenol, 2,5-bis(1,1-dimethyleth, …	206.3	C_14_H_22_O	Phenol
11	10.71	0.01	Octacosane	394.8	C_28_H_58_	Ali. hydrocarbon
12	11.30	0.03	1-Hexadecene	224.4	C_16_H_32_	Ali. hydrocarbon
13	11.38	0.06	Hexadecane	226.4	C_16_H_34_	Ali. hydrocarbon
14	11.93	0.05	2-Undecene, 5-methyl-	168.32	C_12_H_24_	Ali. hydrocarbon
15	12.08	0.02	Hexadecane, 2-methyl-	240.5	C_17_H_36_	Ali. hydrocarbon
16	12.18	0.03	Pentadecane	212.4	C_15_H_32_	Ali. hydrocarbon
17	12.49	0.16	Heptadecane	240.5	C_17_H_36_	Ali. hydrocarbon
18	12.56	0.08	Pentadecane, 2,6,10,14-tetramet, …	268.5	C_19_H_40_	Ali. hydrocarbon
19	12.63	0.04	Hentriacontane	436.8	C_31_H_64_	Ali. hydrocarbon
20	12.97	0.05	Tetratetracontane	619.2	C_44_H_90_	Ali. hydrocarbon
21	13.18	0.04	Heptadecane, 2-methyl-	215.4	C_18_H_38_	Ali. hydrocarbon
22	13.27	0.03	Heptadecane, 3-methyl-	254.9	C_18_H_38_	Ali. hydrocarbon
23	13.51	0.03	1-Octadecene	252.6	C_18_H_36_	Ali. hydrocarbon
24	13.70	0.14	Hexadecane, 2,6,10,14- phytane)	282.5	C_20_H_42_	Diterpene
25	14.12	0.02	7-Oxabicyclo [4.1.0]heptane, 1,5, …	194.2	C_12_H_18_O_2_	Ali. hydrocarbon
26	14.16	0.03	Tetradecane, 5-methyl-	212.4	C_15_H_32_	Ali. hydrocarbon
27	14.24	0.02	Pentadecane	212.4	C_15_H_32_	Ali. hydrocarbon
28	14.54	0.05	Tetrapentacontane, 1,54-dibromo-	917.2	C_54_H_108_Br_2_	Ali. hydrocarbon
29	14.66	0.05	Nonadecane, 9-methyl-	282.5	C_20_H_42_	Ali. hydrocarbon
30	14.84	0.02	Cyclotetradecane, 1,7,11-trimet, …	280.5	C_20_H_40_	Diterpene
31	15.07	0.13	Pentadecanoic acid, 14-methyl-, …	256.4	C_16_H_32_O_2_	Fatty acid
32	15.12	0.02	7,9-Di-tert-butyl-1-oxaspiro(4, …	276.4	C_17_H_24_O_3_	Flavanoids
33	15.36	0.02	Octadecane, 1-chloro-	288.9	C_18_H_37_Cl	Alkyl chloride
34	15.58	0.02	Cyclopentadecane	210.4	C_15_H_30_	Alkane
35	15.90	0.03	1-Nonadecene	266.5	C_19_H_38_	Ali. hydrocarbon
36	16.48	0.30	Heneicosane	296.6	C_21_H_44_	Ali. hydrocarbon
37	16.73	0.04	Octadecane	254.5	C_18_H_38_	Ali. hydrocarbon
38	16.91	0.08	Nonadecane	268.5	C_19_H_40_	Ali. hydrocarbon
39	16.98	0.11	Cycloeicosane	280.5	C_20_H_40_	Alkane
40	17.46	0.14	1-Docosene	308.6	C_22_H_44_	Ali. hydrocarbon
41	17.55	0.12	2-Eicosanol, (.^+^/^−^.)-	298.5	C_20_H_42_O	Phenol
42	17.69	0.16	tert-Hexadecanethiol	258.2	C_16_H_34_S	Thiol
43	18.39	0.17	Tridecane, 6-cyclohexyl-	266.5	C_19_H_38_	Ar. hydrocarbon
44	18.50	0.21	Hexadecanoic acid, butyl ester	312.5	C_20_H_40_O_2_	Fatty acid ester
45	19.19	0.72	Nonahexacontanoic acid	999.8	C_69_H_138_O_2_	Fatty acid
46	19.31	0.19	Nonadecane, 1-chloro-	303	C_19_H_39_Cl	Alkane
47	19.97	0.24	Docosane	310.6	C_22_H_46_	Alkane
48	20.22	0.33	Tricosane	324.6	C_23_H_48_	Alkane
49	20.28	0.23	Cyclotetradecane, 1,7,11-trimet, …	280.5	C_20_H_40_	Alkane
50	20.35	0.32	Nonadecane, 1-chloro-	302.9	C_19_H_39_Cl	Alkane
51	20.43	0.40	1-Chloroeicosane	317.0	C_20_H_41_Cl	Alkyl halide
52	20.61	0.81	Docosane	310.6	C_22_H_46_	Alkane
53	20.69	0.24	Octadecane	254.5	C_18_H_38_	Alkane
54	21.41	0.21	Hexadecane, 1-iodo-	352.34	C_16_H_33_I	Alkyl halide
55	21.64	1.01	1-Chloroeicosane	317.0	C_20_H_41_Cl	Alkyl halide
56	21.80	0.38	1-Tricosene	322.6	C_23_H_46_	Alkene
57	21.91	0.74	1-Nonadecene	266.5	C_19_H_38_	Alkene
58	22.03	1.06	Hexadecane, 1-iodo-	352.34	C_16_H_33_I	Alkyl Halide
59	22.66	1.01	1-Hexacosene	364.7	C _26_H_52_	Alkene
60	22.95	0.99	Pentacosane	352.7	C_25_H_52_	Alkane
61	23.07	1.24	Hexacosane	366.71	C_26_H_54_	Alkane
62	23.45	1.46	Nonadecane, 9-methyl-	282.5	C_20_H_42_	Alkane
63	23.54	0.45	Hexadecane, 2-methyl-	240.5	C_17_H_36_	Alkane
64	23.71	1.05	Di-n-octyl phthalate	390.6	C_24_H_38_O_4_	Benzoic acid esters
65	24.05	0.32	Nonahexacontanoic acid	999.8	C_69_H_138_O_2_	Fatty acid
66	24.12	0.46	Ethanol, 2-(octadecyloxy)-	314.5	C_20_H_42_O_2_	Phenol
67	24.19	0.26	1-Chloroeicosane	317.0	C_20_H_41_Cl	Alkyl halide
68	24.26	1.01	Octadecane	254.4	C_18_H_38_	Alkane
69	24.73	0.77	1-Decanol, 2-hexyl-	242.44	C_16_H_34_O	Alchohol
70	24.85	1.41	Nonadecane, 9-methyl-	282.5	C_20_H_42_	Alkane
71	24.91	0.79	Octadecane, 1-iodo-	380.4	C_18_H_37_I	Alkyl halide
72	26.12	0.74	Tricosane	324.6	C_23_H_48_	Alkane
73	26.24	2.61	Heptacosane, 1-chloro-	415.2	C_27_H_55_Cl	Alkyl halide
74	27.21	1.36	Heptacosane	380.7	C_27_H_56_	Alkane
75	27.58	2.56	Octacosane	394.7	C_28_H_58_	Alkane
76	28.89	3.64	Eicosane	282.5	C_20_H_42_	Ali. hydrocarbon
77	31.40	2.01	Heneicosane, 3-methyl-	310.6	C_22_H_46_	Alkane
78	33.24	0.45	1-Bromoeicosane	361.4	C_20_H_41_Br	Alkyl Halide
79	34.60	0.09	Z-14-Nonacosane	406.8	C_29_H_58_	Alkanes
80	35.49	0.08	Methoxyacetic acid, heptadecyl, …	314.5	C_19_H_38_O_3_	Ester
81	36.25	0.11	Tetratriacontane, 17-hexadecyl-	703.3	C_50_H_102_	Alkanes

**Table 3:** LC-ESI-MS-MS screening of *Strobilanthes glutinosus* in negative mode.

Sr.	RT (min)	% Area	Tentative Identification	Mol. Formula	Mol. Mass	Adduct	Chemical Class
1	10.95	0.49	Aesculetin	C_9_H_6_O_4_	177	[−H]	Coumarin
2	11.70	0.22	Echinospine	C_10_H_9_NO	159	[−H]	Other
3	11.91	1.57	Syringic acid	C_9_H_10_O_5_	199	[−H]	Phenol
4	12.25	0.36	Daidzein	C_15_H_10_O_4_	253	[−H]	Flavonoid
5	12.46	5.84	Hispidulin	C_16_H_12_O_6_	255	[−H]	Flavonoid
6	12.70	0.47	Emmotin A	C_16_H_22_O_4_	277	[−H]	Terpenoid
7	2.21	0.55	p-coumaryl malic acid	C_13_ H_12_ O_7_	279	[−H]	Phenol
8	13.06	2.74	Oleic acid	C_18_H_34_O_2_	281	[−H]	Fatty acid
9	2.37	0.33	Catechin	C_15_H_14_O_6_	289.5	[−H]	Phenol
10	13.32	1.15	Gingerol	C_17_C_26_O_4_	293.50	[−H]	Phenol
11	2.63	0.25	8-Prenylnaringenin	C_20_H_20_O_5_	295.00	[HCOO]	Flavonoid
12	13.40	0.68	Caffeoyl tartaric acid	C_13_H_12_ O_9_	311.00	[−H]	Phenolic acid
13	13.61	0.94	*p*-coumaric acid hexoside	C_15_H_18_O_8_	325.00	[−H]	Phenolic acid
14	14.09	1.28	Sesamolinol	C_20_H_20_O_7_	371.00	[−H]	Lignan
15	14.29	1.16	Oleuropein aglycone	C_19_H_20_O_8_	377	[−H]	Phenol
16	14.49	0.93	Campesterol	C_28_H_48_O	400	[−H]	Terpenoid
17	14.71	1.58	Beta-amyrin	C_30_H_50_O	425	[−H]	Terpenoid
18	15.01	0.33	(−)-Epicatechin 3-O-gallate	C_22_H_18_O_10_	441.50	[−H]	Flavonoid
19	15.99	1.88	Myricetin 3-O-arabinoside	C_20_H_18_O_12_	449.50	[−H]	Flavonoid
20	16.16	1.46	3-Hydroxyphloretin 2′-O-glucoside	C_21_H_24_O_11_	451.15	[−H]	Glucoside
21	16.34	4.32	Lariciresinol-sesquilignan	C_30_H_36_O_10_	555.00	[−H]	Lignan
22	16.76	1.76	Pratensein 7-*O*-β-d-glucoside 6″O-malonate	C_25_H_23_O_13_	549.50	[−H]	Flavonoid
23	17.05	0.49	Luteolin 7- rutinoside	C_27_H_30_O_15_	593	[−H]	Flavonoid

**Table 4:** LC-ESI-MS-MS screening of *Strobilanthes glutinosus* in positive mode.

Sr.	RT (min)	% Area	Tentative Identification	Mol. Formula	Mol. Mass	Adduct	Chemical Class
1	1.50	1.36	Betaine	C_5_H_11_NO_2_	118.00	[+H]	Amino acid
2	2.28	0.94	Gentesic acid	C_7_H_6_O_4_	156.00	[+H]	Phenolic acid
3	2.71	1.44	Azelaic acid	C_9_H_16_O_4_	189.00	[+H]	Dicarboxylic acid
4	3.06	2.57	Angustifoline	C_14_H_22_N_2_O	235.00	[+H]	Alkaloid
5	0.57	0.66	Apigenin	C_15_H_10_O_5_	271.00	[+H]	Flavonoid
6	0.95	0.35	Linoleinic acid	C_18_H_30_O_2_	279.17	[+H]	Fatty acid
7	1.24	0.62	Linoleic acid	C_18_H_32_O_2_	281.50	[+H]	Fatty acid
8	3.43	0.68	Eriodictyol	C_15_H_12_O_6_	289.00	[+H]	Flavonoid
9	3.60	0.44	Catechin	C_15_H_14_O_6_	291.00	[+H]	Phenol
10	3.98	0.27	Gallic acid hexoside	C_13_H_16_O_10_	331.50	[+H]	Phenolic glycoside
11	4.28	0.38	7dehydro cholesterin	C_27_H_44_O	385	[+H]	Terpenoid
12	5.90	1.68	α-tocopherol	C_29_H_50_O_2_	429.30	[+H]	Terpenoid
13	6.21	1.74	5-OH liquiritin	C_21_H_22_O_10_	435.30	[+H]	Flavonoid
14	6.99	2.89	Ligstroside	C_25_H_32_O_12_	523.30	[+H]	Phenolic glycoside
15	6.42	2.33	Scutellarin	C_21_H_18_O_12_	463.30	[+H]	Flavonoid
16	7.33	1.46	Agnuside	C_22_H_26_O_11_	467.50	[+H]	Other
17	7.57	5.18	di-O-acetyldarutoside	C_30_H_48_O_10_	567.50	[+H]	Phenol
18	7.87	4.78	Rutin	C_27_H_30_O_16_	611.20	[+H]	Flavonoid
19	8.32	4.56	Quercetin-6,4′-dimethoxy-3-fructo-rhamnoside	C_21_H_20_O_11_	655.50	[+H]	Flavonoid
20	8.84	7.23	Quercetin rhamnoside-feruloyl-hexoside	C_31_H_28_O_15_	743.55	[+H]	Flavonoid
21	9.97	5.99	Quercetin 3-*O*-rhamnosyl-glucoside 7-*O*-rhamnoside	C_27_H_30_O_16_	875.50	[+H]	Flavonoid

**Table 5:** Antioxidant results by different methods of *S. glutinosus* whole plant extract/fractions.

Extract/Fractions	Radical Scavenging Assays		Reducing Power Assays		Total Antioxidant Capacity	Ferrous Ion Chelation
	DPPH (mg TE/g Extract)	ABTS (mg TE/g Extract)	CUPRAC (mg TE/g Extract)	FRAP (mg TE/g Extract)	Phosphomolybdenum (mg TE/g Extract)	Metal Chelation (mg EDTAE/g Extract)
Methanol	56.217 ± 0.66 ^a^	63.469 ± 0.045 ^a^	245.116 ± 4.240 ^a^	87.126 ± 0.083 ^a^	96.015 ± 0.476 ^b^	17.038 ± 0.0769 ^b^
*n*-butanol	47.920 ± 0.166 ^c^	47.669 ± 0.078 ^c^	162.629 ± 6.372 ^b^	75.097 ± 0.054 ^b^	6.544 ± 0.748 ^d^	7.692 ± 0.0769 ^d^
Chloroform	50.130 ± 0.108 ^b^	53.574 ± 5.183 ^b^	84.693 ± 2.780 ^c^	65.007 ± 0.361 ^c^	60.698 ± 0.079 ^c^	12.384 ± 8.423 ^c^
*n*-hexane	9.804 ± 1.234 ^d^	12.761 ± 0.045 ^d^	59.878 ± 4.865 ^d^	34.971 ± 1.820 ^d^	119.587 ± 0.555 ^a^	25.346 ± 0.192 ^a^

All of the procedures were carried out thrice. The mean ± standard deviation were used to represent the results. Trolox and EDTAE were utilized as standard. Significantly different results were exhibited when compared to standard (*p* < 0.05). Superscripts (a–d) represents statistical difference.

**Table 6:** Enzyme inhibition results of *S. glutinosus* whole plant extract/fractions.

Extract/Fractions	α-Amylase (mmol ACAE/g Extract)	α-Glucosidase (mmol ACAE/g Extract)	Tyrosinase (mg KAE/g Extract)
Methanol	166.758 ± 1.72 ^b^	294.195 ± 3.036 ^b^	9.86 ± 1.4 ^a^
*n*-butanol	107.007 ± 1.104 ^c^	118.64 ± 1.224 ^c^	8.52 ± 1.82 ^b^
Chloroform	501.407 ± 2.982 ^a^	605.854 ± 6.252 ^a^	6.91 ± 1.35 ^c^
*n*-hexane	85.859 ± 0.510 ^d^	93.572 ± 0.965 ^d^	NA

All of the procedures were carried out thrice. The mean ± standard deviation were used to represent the results. ACAE: acarbose equivalent, KAE: kojic acid equivalent. Significantly different results were exhibited when compared to standard (*p* < 0.05). NA (No Activity). Superscripts (a–d) represents statistical difference.

**Table 7:** Binding affinities and interactions of ligands against (anti-diabetic) enzymes.

Enzyme	Ligands	Binding Energy (kcal/mol)	Electrostatic/Hydrophobic Interaction
α-glucosidase	Acarbose	−6.6	Hydrogen bond (Thr^448^, Asn^443^, Ala^514^, Asp^441^, Glu^432^, Arg^437^, His^348^) C-H bond (Gln^438^)
	Lupeol	−6.9	Alkyl interaction (Lys^398^, Trp^394^, Val^380^, Trp^354^) Hydrogen bond (Ala^229^, Asn^301^) Van-dar walls (Pro^230^, Arg^340^, Phe^357^, Ala^378^, Gly^402^, Glu^377^, Val^335^, Leu^227^, Met^302^, Glu^396^, Asp^379^, Glu^231^)
	Sitosterol	−7.5	Alkyl interaction (Leu45, Ala444) C-H bond (Leu433) Van-dar walls (Ala434, Arg450, Met407, Thr445)
	9,12-octadecadienoic acid	−4.1	Alkyl interaction (Leu^446^) Van-dar walls (Asp^411^, Thr^410^, Leu^373^, Leu^45^, Asp^440^, Ser^44^, Gln^438^, Pro^408^, Glu^432^, Leu^431^)
	β-Amyrin	−8.4	Alkyl interaction (Pro^230^)
	Hexadacanoic acid	−3.9	Hydrogen bond (Aal^380^) C-H bond (Lyc^398^, Gly^998^) Alkyl interaction (Val^335^)
	Stigmasterol	−7.5	Alkyl interaction (Val^335^, Ala^343^, Met^302^, Val^334^, Phe^397^, Phe^297^)
α-amylase	Lupeol	−7.6	Pi-sigma (Tyr^59^) Pi-alkyl (Trp^60^)
	Sitosterol	−5.1	Pi-alkyl (Pro^230^, Phe^397^)
	9,12-octadecadienoic acid	−4.9	Hydrogen bond (Asn^273^) Alkyl interaction (Tyr^59^, Leu^142^, Met^302^, Ala^177^, Leu^141^)
	β-Amyrin	−8.4	Hydrogen bond (Asp^274^) Pi-sigma (Phe^105^) Pi-alkyl (Tyr^59^)
	Hexadacanoic acid	−4.6	Van-dar walls (Asp^274^, Tyr^62^, Ala^177^, Asp^176^, Gln^63^, Asp^269^, Leu^144^, Asn^273^) Pi-alkyl (Tyr^59^, Trp^58^, Leu^142^) C-H bond (His^102^, Gln^208^)
	Stigmasterol	−9.1	Pi-sigma (Tyr^59^) Pi-alkyl (Tyr^59^, Leu^142^, Phe^105^)

**Table 8:** Binding affinities and interactions of the selected ligands from *S. glutinosus* extract by LC-ESI-MS against tyrosinase enzyme.

Enzyme	Ligand	Binding Affinity (Kcal/mol)	Amino Acids Interactions
Tyrosinase	Ligstroside	−8.0	Unfavorable Accaptor: (TYR^A352^) Pi Sigma: (VAL^A366^) Conventional Hydrogen Bond: (ASN^A15^, GLN^A294^, GLY^A360^, GLY^A361^) Carbon Hydrogen: (SER^A351^, PHE^A355^)
	Rutin	−8.9	Amide-Pi Stacked: (PHE^A355^) Pi-Alkyl: (PRO^A298^, LYS^A359^) Conventional Hydrogen Bond: (GLN^A294^, THR^A345^, VAL^A358^, GLY^A360^, GLY^A361^) Van der Waals: (VAL^A13^, GLY^A299^, VAL^A300^, THR^A343^, ASP^A344^, ALA^A346^, SER^A351^, TYR^A352^, PRO^A363^, VAL^A366^)
	Scutellarin	−8.6	Pi-Pi Stacked: (PHE^A355^) Pi-Alkyl: (ALA^A295^, PRO^A298^) Conventional Hydrogen Bond: (GLN^A294^) Carbon Hydrogen: (THR^A343^) Van der Waals: (SER^A291^, TYR^A297^, GLY^A299^, VAL^A300^, TRP^A301^, THR^A345^, SER^A351^, TYR^A352^, PRO^A363^, VAL^A366^)
	Kojic acid (Standard)	−5.3	Pi-Pi Stacked: (PHE^A355^)

## Data Availability

Not applicable.

## Supplementary Materials

The following supporting information can be downloaded at: https://www.mdpi.com/article/10.3390/molecules27206885/s1, Table S1: List of abbreviations; Table S2: Pharmacological properties of various species of the genus *Strobilanthes* [40,41,42,43,44,45,46,47,48,49,50].
