# Supplementary material for: Comprehensive Biological Potential, Phytochemical Profiling Using GC-MS and LC-ESI-MS, and In-Silico Assessment of Strobilanthes glutinosus Nees: An Important Medicinal Plant
Source: Molecules. 2022 Oct 14;27(20):6885. doi: 10.3390/molecules27206885 (PMC9608989; doi:10.3390/molecules27206885)
Supplement: Supplementary file 1 [file molecules-27-06885-s001.zip › molecules-1942895-SM.pdf]

**Table S1.** List of abbreviations

| Sr. | Abbreviations | Full form |
|---|---|---|
| 1 | DPPH | 2,2-diphenyl-1-picryl-hydrazyl-hydrate |
| 2 | ABTS | 2,2'-azino-bis(3-ethylbenzothiazoline-6-sulfonic acid |
| 3 | CUPRAC | cupric reducing antioxidant capacity |
| 4 | FRAP | Ferric Reducing Antioxidant Power |
| 5 | GC-MS | Gas chromatography–mass spectrometry |
| 6 | LC-ESI-MS | Liquid Chromatography-Electrospray Ionization-Mass Spectrometry |
| 7 | ACAE | Acarbose Equivalent |
| 8 | KAE | Kojic acid Equivalent |
| 9 | TE | Trolox Equivalent |
| 10 | RT | Retention Time |

**Table S2.** Pharmacological properties of various species of the genus Strobilanthes

| Sr. | Specie Name | Pharmacological activity | Reference |
|---|---|---|---|
| 1 | *Strobilanthes cuspidate* | Anti-inflammatory, antidiabetic | [1] |
| 2 | *Strobilanthes cusia* | Antipyretic, analgesic, anti-inflammatory, antiviral, antimicrobial, meningitis, encephalitis, mumps, | [2-4] |
| 3 | *Strobilanthes callosus* | Anti-inflammatory, antibacterial, analgesic, and anti arthritic | [5-7] |
| 4 | *Strobilanthes ixiocephala* | Anti-inflammatory and anti arthritic activities | [5] |
| 5 | *Strobilanthes crispus* | Antioxidant, antidiabetic, antibacterial, anticancer, and antihyperlipidemic | [8-11] |
| 6 | *Strobilanthes kunthianus* | Antioxidant activity and hepatoprotective | [12] |
| 7 | *Strobilanthes glutinosus* | Antioxidant and antimicrobial | [13] |

**References.**


1. Joseph, S., L. Kumar, and V.N. Bai, *Evaluation of anti-diabetic activity of Strobilanthes cuspidata in alloxan induced diabetic rats and the effect of bioactive compounds on inhibition of [alpha]-amylase enzyme.* Journal of Pharmacognosy and Phytochemistry, 2016. **5**(3): p. 169.
2. Ho, Y.-L., et al., *Evaluation of antinociceptive, anti-inflammatory and antipyretic effects of Strobilanthes cusia leaf extract in male mice and rats.* The American journal of Chinese medicine, 2003. **31**(01): p. 61-69.
3. Mitscher, L.A. and W. Baker, *Tuberculosis: a search for novel therapy starting with natural products.* Medicinal research reviews, 1998. **18**(6): p. 363-374.
4. Tsai, Y.-C., et al., *Antiviral action of tryptanthrin isolated from Strobilanthes cusia leaf against human coronavirus NL63.* Biomolecules, 2020. **10**(3): p. 366.
5. Agarwal, R. and V. Rangari, *Antiinflammatory and antiarthritic activities of lupeol and 19 alpha-H lupeol isolated from Strobilanthus callosus and Strobilanthus ixiocephala roots.* Indian Journal of Pharmacology, 2003. **35**(6): p. 384-387.
6. Kumar, S., et al., *Acute and chronic inflammation studies of Strobilanthes callosus leaves extract on rat model.* Inflammopharmacology, 2013. **21**(3): p. 233-239.
7. Singh, B., P. Sahu, and M. Sharma, *Anti-inflammatory and antimicrobial activities of triterpenoids from Strobilanthes callosus Nees.* Phytomedicine, 2002. **9**(4): p. 355-359.
8. Abou Muamar, A.F.H., *Isolation, Identification and Evaluation of Antibacterial Activity of the Semi-purified Compound from Strobilanthes Crispus (L. Bremek)*. 1999, Universiti Putra Malaysia.
9. Bakar, M.F.A., *Effects of Strobilanthes Crispus Crude and Tea Extracts in Streptozotocin-induced Hyperglycemic Rats*. 2005, Universiti Putra Malaysia.
10. Fadzelly, A., R. Asmah, and O. Fauziah, *Effects of Strobilanthes crispus tea aqueous extracts on glucose and lipid profile in normal and streptozotocin-induced hyperglycemic rats.* Plant Foods for Human Nutrition, 2006. **61**(1): p. 6-11.
11. Ghasemzadeh, A., H.Z. Jaafar, and A. Rahmat, *Phytochemical constituents and biological activities of different extracts of Strobilanthes crispus (L.) Bremek leaves grown in different locations of Malaysia.* BMC complementary and alternative medicine, 2015. **15**(1): p. 1-10.
12. Balasubramaniam, G., et al., *Antioxidant and hepatoprotective activities of Strobilanthes kunthianus against carbon tetrachloride-induced hepatotoxicity in rats.* Pharmacognosy Journal, 2020. **12**(5).
13. Ajaib, M., et al., *Antimicrobial and antioxidant analysis of Strobilanthes glutinous: an unexplored medicinal plant.* Bioscience Research, 2020. **17**(2): p. 1521-1534.
